# Diffuse Large B-Cell Lymphoma Promotes Endothelial-to-Mesenchymal Transition *via* WNT10A/Beta-Catenin/Snail Signaling

**DOI:** 10.3389/fonc.2022.871788

**Published:** 2022-04-12

**Authors:** Xianting Sun, Jianchen Fang, Fen Ye, Shuxian Zhang, Honghui Huang, Jian Hou, Ting Wang

**Affiliations:** ^1^ Department of Hematology, Renji Hospital, School of Medicine, Shanghai Jiaotong University, Shanghai, China; ^2^ Department of Pathology, Renji Hospital, School of Medicine, Shanghai Jiaotong University, Shanghai, China

**Keywords:** angiogenesis, diffuse large B-cell lymphoma, endothelial-to-mesenchymal transition, WNT/β-catenin, snail

## Abstract

Diffuse large B-cell lymphoma (DLBCL) is one type of highly heterogeneous lymphoid malignancy with 30%~40% of patients experiencing treatment failure. Novel risk stratification and therapeutic approaches for DLBCL are urgently needed. Endothelial-to-mesenchymal transition (EndMT), which contributes to tumor angiogenesis, metastasis, drug resistance, and cancer-associated fibroblast generation, has been detected in the microenvironment of many types of cancers. However, the existence of EndMT in the hematological malignancies microenvironment remains unknown. Here, we identified the existence of EndMT in DLBCL-associated endothelial cells and the clinical relevance of EndMT markers in DLBCL, which was associated with advanced clinical stage and poor prognosis. *In vitro* experiments confirmed that DLBCL cells stimulated angiogenesis and EndMT of human umbilical vein endothelial cells (HUVECs). We further unveiled the molecular mechanisms underlying this process. We demonstrated that WNT10A, a WNT family member overexpressed in DLBCL tissues and correlated with clinical features in DLBCL, promoted EndMT through glycogen synthase kinase 3β (GSK3β)/β-catenin/snail signaling. WNT10A inhibited the binding of GSK3β to β-catenin/snail, resulting in β-catenin and snail nuclear accumulation and target gene transcription. Silencing β-catenin and snail respectively attenuated WNT10A-induced angiogenesis and EndMT. The interplay between β-catenin-dependent and snail-dependent signaling was also confirmed in this study. Collectively, these findings identified that WNT10A/GSK3β/β-catenin/snail pathway performed vital roles in DLBCL-induced EndMT and indicated that EndMT markers and WNT10A may serve as novel predictors of clinical outcome.

## Introduction

Diffuse large B-cell lymphoma (DLBCL), the most common adult lymphoid malignancy, is highly genetically and clinically heterogeneous (1). Patients are differentially characterized by involved organs, therapeutic efficacy, and prognosis. Though the overall remission rate has been significantly increased along with the application of rituximab, 30%~40% of DLBCL patients suffered from primary refractory or disease progression (2). Therefore, it is essential to explore new markers for DLBCL early detection, risk stratification, and prognosis prediction. In-depth studies of the molecular mechanisms underlying DLBCL progression and novel therapeutic approaches were also urgently needed.

Endothelial-to-mesenchymal transition (EndMT) is a type of cellular transdifferentiation in which endothelial cells revealed substantial plasticity such as cytoskeleton rearrangement, loss of intercellular tight conjunction, and acquisition of migratory potential. During this process, endothelial cells downregulate endothelial markers including CD31 and vascular-endothelial cadherin (VE-cadherin) and acquire mesenchymal markers such as α-smooth muscle actin (α-SMA), fibronectin (FN), vimentin, collagen type I (COLI), and serpine1 (3). EndMT has been detected and been proved to play a pivotal role during embryonic development as well as pathologies such as malignancy, fibrosis, and vascular diseases. During malignancy, EndMT was a source of cancer-associated fibroblasts, which can facilitate tumor growth through extracellular matrix (ECM) remodeling, growth factor production, and immunoregulation (4, 5). EndMT can also promote angiogenesis, cancer metastasis, and therapy resistance (6, 7). Nevertheless, the existence of EndMT in DLBCL and its role in DLBCL progression remain elusive.

Numerous signaling pathways (including TGF-β, wingless-related integration site (WNT), endothelin-1, hypoxia-inducible factor-1, and Notch pathway) and transcription factors (including SNAI1 (snail), ZEB1, ZEB2, and TWIST1) participate in the EndMT process (8). WNT signaling is aberrantly activated in plenty of tumors including lymphoma and contributes to the development of cancer (9, 10). WNT ligands attenuate GSK3β activity through the interaction between WNT receptor and GSK3β, which prevents the phosphorylation of β-catenin and facilitates β-catenin cytoplasmic accumulation, nucleus translocation, and target gene transcription (11–13). Apart from β-catenin, several dozen proteins are also reported to function as the phosphorylation substrates of GSK3β and regulated by WNT. This process is referred to as WNT-dependent stabilization of proteins (WNT/STOP) now (12, 14). Snail, a master transcription factor in EndMT, differs from the other transcription factors. Unlike the other transcription factors, snail also plays a crucial role in angiogenesis and is one of the Wnt/STOP target proteins (15).

WNT family comprises 19 secreted glycoproteins implicated in both embryogenesis and virtually every adult tissue hemostasis (16–18). WNT10A, one member of the WNT family, has been previously elucidated in ectodermal disorders (19). Mutation of WNT10A may lead to abnormal tooth development and kinds of skin alterations (19, 20). In addition, a series of studies have reported that WNT10A was upregulated in colorectal cancer and esophageal cancer. Overexpression of WNT10A was associated with cancer invasion as well as poor prognosis *via* regulation of β-catenin (21, 22). Yet the role of WNT10A in EndMT and hematological malignancy progression remains elusive.

Based on these previous studies, we detected the presence of EndMT in the supportive tissues of DLBCL. Our investigation identified that WNT10A was exceptionally overexpressed in DLBCL and played a pivotal role in DLBCL-induced angiogenesis and EndMT. Mechanistically, we observed that WNT10A blunted GSK3β activity, thereby reducing its interaction with β-catenin and snail. Increased accumulation of β-catenin and snail in the nucleus subsequently upregulated the expression of genes triggering EndMT and angiogenesis.

## Materials and Methods

### Cell Culture and Reagents

Human umbilical vein endothelial cells (HUVECs) and DLBCL cell line OCI-LY3 were purchased from American Type Culture Collection (ATCC, Manassas, VA, USA). DLBCL cell line SU-DHL-4 was kindly provided by Stem Cell Bank, Chinese Academy of Science. Cells were maintained in high-glucose Dulbecco’s modified Eagle’s medium (DMEM) (Gibco, Grand Island, NY, USA), IMDM (Gibco, USA), and Roswell Park Memorial Institute (RPMI)/1640 (Gibco, USA) supplemented with 10% fetal bovine serum (FBS, Gibco). Recombinant human WNT10A (rhWNT10A) was purchased from Cloud-Clone Corp. (Wuhan, China). IWR-1 was purchased from Sigma (St. Louis, MO, USA). Antibodies against the following proteins were purchased from Abcam (Boston, MA, USA): WNT10A (ab106522), snail (ab216347), CD31 (ab9498), vimentin ab (ab8978), α-SMA (ab7817), Lamin B (ab16048), and IL-1β (ab234437). Antibodies against GSK3β (#12456), β-catenin (#8480), total/phospho-p65 (#8242, #3033), total/phospho-IkB-α (#4814, #2859), β-actin (#3700), and VE-cadherin (#2500) were purchased from Cell Signaling Technology (Danvers, MA, USA). Antibodies against FN (CY5621), ICAM-1 (CY5391), VCAM-1 (CY5427), COLI (CY5120), anti-COX-2 (CY5580), and GAPDH (AB0036) were purchased from Abways Technology (Shanghai, China). Antibodies for immunoblot and immunoprecipitation were used at a dilution of 1:1,000 and 1:50, respectively, while antibodies for immunohistochemistry and immunofluorescence were used at a dilution of 1:100.

### Co-Culture Model

In this study, an indirect two-layer co-culture model was established to mimic the tumor microenvironment. HUVECs cultured in the lower chamber were incubated with DLBCL cells in the upper chamber and were harvested at indicated time points for further analyses. HUVECs and DLBCL cells were separated by the transwell chamber (pore size 0.4 μm, Corning, New York, NY, USA), while they can interact with each other through the medium, which could diffuse freely across the membrane.

### Immunohistochemistry and Immunofluorescence Staining

For tissue immunofluorescence and immunohistochemistry, paraffin-embedded human tissues were de-paraffinized and underwent antigen retrieval in citrate antigen retrieval solution at 95°C for 20 min. Sections were then blocked by 3% bovine serum albumin (BSA) for 30 min and incubated with primary antibody overnight at 4°C. Secondary antibodies were added on the next day. For immunofluorescence, the nucleus was counterstained with DAPI solution, and slides were imaged with Z-stack using confocal microscopy (Eclipse Ti, NIKON, Melville, NY, USA). For immunohistochemistry, objective tissue was covered with DAB color-developing solution, and the nucleus was counterstained with hematoxylin stain solution. Slides were scanned with Pannoramic 250FLASH (3DHISTECH, Budapest, Hungary). Three random fields were selected per sample for quantified analysis.

For cell immunofluorescence staining, cells were cultivated on glass coverslips. Cells were fixed with 4% polyformaldehyde, permeabilized with 0.2% Triton X-100, and blocked with 5% BSA, followed by incubation with primary antibodies at 4°C overnight. On the next day, after incubation with secondary antibodies for 1 h, 3 random fields per coverslip were selected and imaged by fluorescence microscopy for further analysis.

For the WNT10A assessment, stained sections were scored by two pathologists in a blind manner. The staining intensity was scored as 0 (negative), 1 (weak), 2 (median), and 3 (strong). The extent of staining was scored as 0 (<5%), 1 (5%–25%), 2 (26%–50%), 3 (51%–75%), and 4 (76%–100%). The final staining index (SI) was determined by intensity score × staining score. SI < 3 was defined as low expression, while SI ≥ 4 was defined as a high expression.

### Immunoblot Analysis and Co-Immunoprecipitation Assay

Total cell protein was extracted using radioimmunoprecipitation assay (RIPA) lysis buffer (Beyotime, Shanghai, China) with proteasome inhibitor cocktail (Sigma, USA). Nuclear and cytoplasmic proteins were extracted using Nuclear and Cytosol Fractionation Kit (BioVision Inc., Milpitas, CA, USA). In immunoblot analysis, the protein was electrophoresed by sodium dodecyl sulfate–polyacrylamide gel electrophoresis (SDS-PAGE) and transferred to polyvinylidene fluoride membrane (Millipore, Billerica, MA, USA). Membranes were blocked with 5% BSA for 1 h and then incubated at 4°C overnight with primary antibodies. On the next day, membranes were incubated with a secondary antibody for 1 h and detected by enhanced chemiluminescence (ECL).

HUVECs were lysed by NP-40 lysis for subsequent co-immunoprecipitation (Co-IP). Extracted proteins were incubated with the corresponding antibody at 4°C for 2 h and then incubated with protein A/G agarose beads at 4°C overnight. After centrifuging, samples were subjected to immunoblot.

### RNA Isolation and Quantitative Real-Time PCR

RNA was extracted using RNeasy Mini Kit (Qiagen, Hilden, Germany), followed by reverse transcription. Quantitative Real-Time PCR (qRT-PCR) was performed with ChamQ Universal SYBR qPCR Master Mix (Vazyme, Nanjing, China) according to the instruction manuals. The primer sequences used in this study are listed in **Table S1**.

### Cell Permeability

HUVECs were seeded on transwell insert (pore size 0.4 μm; Corning) until they generated an intact monolayer, and then DLBCL cells were added to the lower compartment. After co-culture for 24 h, the medium in the upper compartment was replaced with 1 mg/ml of fluorescein isothiocyanate (FITC)-dextran (molecular weight 70,000 kDa, Sigma, USA) diluted with the medium; 100 μl of the medium was collected from the lower compartment after 60 min, and fluorescence intensity was measured with excitation/emission of 490/520 nm.

### Cell Proliferation Assay

A total of 4,000 HUVECs were seeded into 96-well plates and co-cultured with DLBCL cells or WNT10A. At 24, 48, and 72 h, the medium was removed, and HUVECs were gently washed with warm PBS to remove any adherent DLBCL cells, which was further confirmed under microscopy. Then, 100 μl of culture medium containing 10 μl of Cell Counting Kit-8 (CCK-8) solution (Dojindo Laboratories, Tokyo, Japan) was added. After incubation for 2 h, absorbance at 450 nm was measured by a microplate reader.

### Transwell Migration Assay

Migration assays were performed in a 24-well transwell plate (pore size 8 μm, Corning, USA). Approximately 5 × 10^5^ HUVECs in serum-free medium were seeded on the transwell insert; 600 μl of complete culture medium was added to the lower chamber. After incubation for 24 h, unmigrated cells were carefully wiped with a cotton swab, and migrated cells were fixed with 4% polyformaldehyde. After being washed with PBS, cells were dyed with crystal violet and counted under a microscope.

### Gelatin Zymography

Extracted proteins of HUVECs were separated using SDS-PAGE containing 1 mg/ml of gelatin. Following electrophoresis, gels were washed in buffer containing 2.5% Triton-X 100, 50 mM of Tris-HCl, 5 mM of Cacl_2_, and 1 μM of Zncl_2_ and incubated in buffer containing 50 mM of Tris-HCl, 150 mM of Nacl, 10 mM of Cacl_2_, 1 μM of Zncl_2_, and 0.02% Brij for 48 h. After being stained by Coomassie Blue Staining Solution (Beyotime, China) for 3 h, gels were washed in Coomassie Blue Staining Destaining Solution (Beyotime, China). Images were captured by Gel Doc™ EZ Imager (Bio-Rad, Hercules, CA, USA).

### Matrigel Tube Formation Assay

Pre-cold 96-well plates were coated with 50 μl of pre-thawed Matrigel (BD Biosciences, San Jose, CA, USA) for 1 h at 37°C. The harvested HUVECs were suspended in DMEM with 3% FBS alone or other specific treatments and seeded onto the Matrigel. Tube formation ability was observed and captured at 5 and 9 h. Total tube length and number of nodes were quantified by ImageJ software.

### RNA Interference and Reporter Assay

Transfection was performed with Lipofectamine 3000 (Invitrogen, Carlsbad, CA, USA) according to the manufacturer’s instructions. WNT10A siRNA, β-catenin siRNA, and snail siRNA were designed and synthesized by Genomeditech (Shanghai, China) and were transfected into cells at 75 nM. The knockdown efficacies of siRNAs were confirmed by quantitative real-time PCR (qRT-PCR) and immunoblot, and the results are shown in **Figure S1**. Sequences of siRNAs used in this study are listed in **Table S2**. In luciferase reporter assay, Top/FopFlash reporter construct or β-catenin promoter construct (GeneChem, Shanghai, China) along with pRL-TK Renilla plasmid (Promega, Madison, WI, USA) were co-transduced into HUVECs. After 24 h of transfection, cells were treated with WNT10A for 24 h, and luciferase activity was detected with Dual-Lumi™ II Luciferase Reporter Gene Assay Kit (Beyotime, China). Reporter activity in each group was calculated as the ratio of firefly luciferase intensity to renilla luciferase intensity.

### Statistical Analysis

All experiments were performed three independent times. Quantitative results were expressed by mean ± SD. Two-tailed Student’s *t*-test was used for 2 group comparisons, and one-way ANOVA was used for comparison between multiple groups. Survival curves were plotted by the Kaplan–Meier method, and differences between subgroups were calculated using a log-rank test. *p* < 0.05 was considered to be statistically significant.

## Results

### Endothelial-to-Mesenchymal Transition Was Present in Diffuse Large B-Cell Lymphoma Tissues and Associated With Patient Survival

**Figure 1:**
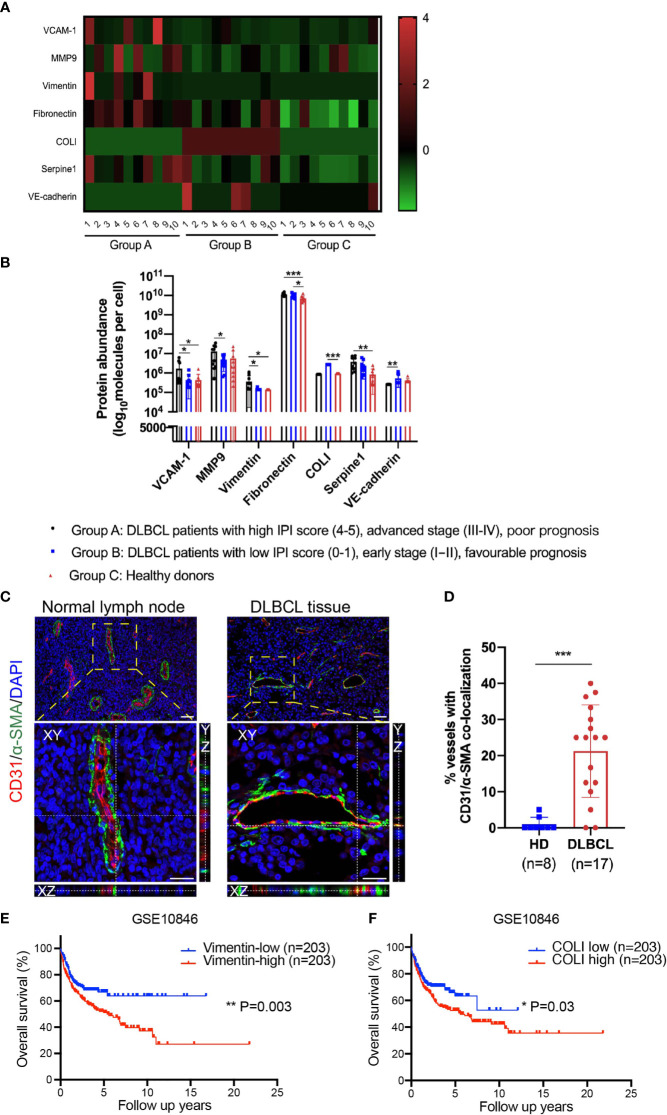
EndMT was detected in DLBCL tissues, and EndMT markers were correlated with poor prognosis. **(A, B)** Proteomics analysis of serum exosomes from DLBCL patients and healthy donors (HD). Group A (n = 10), Group B (n = 10), and Group C (n = 10). **(A)** A heatmap showing differentially expressed EndMT marker proteins. Protein abundance was standardized by Z-score transformation. **(B)** Statistical analysis of differentially expressed EndMT marker proteins between 3 groups. **(C)** Representative orthogonal views of normal lymph nodes and DLBCL tissues double staining with endothelial cell marker CD31 (red) and mesenchymal cell marker α-SMA (green). Endothelial cells underwent EndMT co-expressed CD31/α-SMA, giving rise to yellow staining. Top panel: scale bar, 50 μm; magnification, ×200. Bottom panel: scale bar, 25 μm; magnification, ×400. **(D)** The percentage of vascular endothelial cells displaying CD31/α-SMA co-localization. **(E, F)** Kaplan–Meier analysis showed levels of vimentin and COLI were negatively associated with overall survival in DLBCL. Gene expression profiling data of DLBCL patient samples were obtained from GEO/GSE10846. Error bars, SD. *p < 0.05, **p < 0.01, ***p < 0.001. EndMT, endothelial-to-mesenchymal transition; DLBCL, diffuse large B-cell lymphoma; α-SMA, α-smooth muscle actin; COLI, collagen type I; GEO, Gene Expression Omnibus.

Exosomes were membrane vesicles with an average diameter of 30–100 nm in all biological fluids (23). Serum exosomes containing nucleic acids, proteins, lipids, amino acids provide a pure sample without plasma and can offer useful information for clinical analysis. Our group previously investigated the protein expression profiles of exosomes through label-free quantification proteomics. Serum exosomes were extracted from 20 DLBCL patients and 10 healthy donors who were divided into 3 groups. Group A included 10 DLBCL patients with a high International Prognostic Index (IPI) score (4–5), advanced clinical stage (III–IV), and poor prognosis. Group B included 10 DLBCL patients with low IPI scores (0–1), early clinical stage (IA/IB), and favorable prognosis. Group C included 10 healthy donors. We found endothelial cell marker VE-cadherin was downregulated in group A compared with group B, whereas mesenchymal cell markers vimentin and MMP9, were upregulated in group A compared with group B. Vascular cell adhesion molecule-1 (VCAM-1), an endothelial activation marker, was also upregulated in Group A. In addition, overexpression of mesenchymal cell markers FN, vimentin, COLI, and serpine1 were detected in DLBCL patients compared with healthy donors (**Figures 1A, B**). Though these changes were consistent with the process of EndMT, they are not enough to be an indication of EndMT, as serum exosomes can be secreted by a wide range of cell types (not only endothelial cells), and there are many reasons for the upregulation of these mesenchymal-specific proteins such as excessive secretion by mesenchymal cells. However, these findings do inspire us to figure out whether EndMT is present in DLBCL-associated endothelial cells, which could partially contribute to these changes in serum exosomes.

In the previous authoritative reports, endothelial marker/mesenchymal marker co-localization revealed by double immunofluorescence staining is suggestive of tissue endothelial cells undergoing EndMT (24–26). In this study, paraffin-embedded sections from DLBCL lymph nodes and matched normal lymph nodes were stained with double immunofluorescence for endothelial marker CD31 and mesenchymal cell marker α-SMA. In vessels of normal lymph nodes, as expected, most of the α-SMA expression was around the endothelial layer, while in DLBCL patients, endothelial cells co-expressed CD31/α-SMA (**Figure 1C**). Using confocal imaging with Z-stack, we confirmed CD31/α-SMA co-localization in DLBCL tissues not only in a two-dimensional plane but also in the three-dimensional space, and representative orthogonal views are demonstrated in **Figure 1C**.

Statistically, the percentage of CD31/α-SMA co-localization vessels prominently increased in DLBCL patients (**Figure 1D**). We also performed survival analysis with EndMT markers vimentin, COLI, CD31, VE-cadherin, and FN in DLBCL tissues using Gene Expression Omnibus (GEO) datasets (GSE10846). Consistent with the above results, the Kaplan–Meier plotter analysis demonstrated that high expression levels of vimentin and COLI in DLBCL tissues were correlated with poor overall survival (**Figures 1E, F**). However, CD31, VE-cadherin, and FN showed no relevance to overall survival in this dataset (data not shown).

### Human Umbilical Vein Endothelial Cells Co-Cultured With Diffuse Large B-Cell Lymphoma Cells Have Undergone Endothelial-to-Mesenchymal Transition

**Figure 2:**
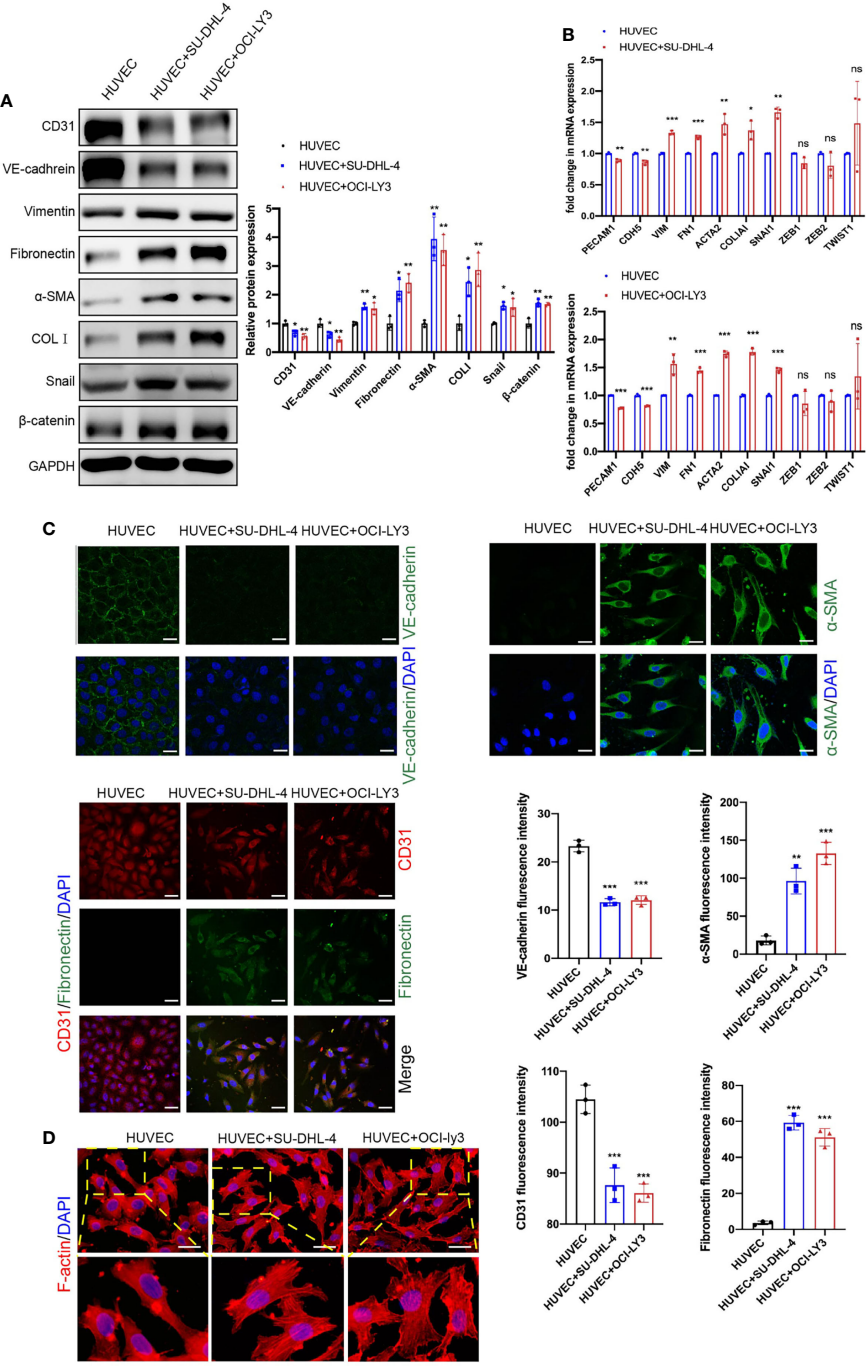
Endothelial cells co-cultured with GCB and ABC DLBCL cells exhibited mesenchymal phenotype. **(A)** Protein expression changes of EndMT markers and β-catenin were detected by immunoblot (n = 3 independent experiments). **(B)** mRNA expression changes of EndMT markers were detected by qRT-PCR (n = 3 independent experiments). **(C)** Representative fluorescence images showing downregulation of endothelial markers VE-cadherin (C1, green) and CD31 (C3, red) and upregulation of mesenchymal cell markers α-SMA (green, C2) and fibronectin (green, C3) in HUVECs co-cultured with DLBCL cells. Nuclei were stained with DAPI. Scale bar, 25 μm; n = 3 independent experiments. **(D)** Representative fluorescence images of skeleton changes in HUVECs by F-actin staining (n = 3 independent experiments). Scale bar, 25 μm. Error bars, SD; *p < 0.05, **p < 0.01, ***p < 0.001 vs. control. ns, not significant; qRT-PCR, quantitative real-time PCR; GCB, germinal center B cell; ABC, activated B cell; DLBCL, diffuse large B-cell lymphoma; EndMT, endothelial-to-mesenchymal transition; HUVEC, human umbilical vein endothelial cell.

To investigate the interaction between ECs and DLBCL cells, we selected two types of DLBCL cell lines, germinal center B cell (GCB; SU-DHL-4) and activated B cell (ABC; OCI-LY3), and we established an indirect co-culture system using a transwell chamber. HUVECs in the lower chamber were co-cultured with DLBCL cells in the upper chamber for 48 h. As displayed in **Figure 2A**, immunoblot assay revealed the reduction of endothelial markers CD31 and VE-cadherin and induction of mesenchymal markers vimentin, FN, α-SMA, and COLI in HUVECs after co-culture with SU-DHL-4 and OCI-LY3 cells (**Figure 2A**). Meanwhile, SU-DHL-4 and OCI-LY3-induced EndMT were further confirmed by qRT-PCR and immunofluorescence staining (**Figures 2B, C**). We also detected the mRNA levels of classical transcription factors including SNAI1, ZEB1, ZEB2, and TWIST1 in HUVECs after co-culture with DLBCL cells for 24 h. The results showed that, among these transcription factors, only the mRNA level of SNAI1 was statistically increased in HUVECs, while no significant increase was observed in the mRNA levels of ZEB1, ZEB2, and TWIST1 (**Figure 2B**). As skeleton rearrangement was a key step involved in EndMT, F-actin filaments in HUVECs were stained by rhodamine phalloidin. Increased lamellipodia, filopodia, and actin bundles were observed in HUVECs co-cultured with SU-DHL-4 or OCI-LY3 cells (**Figure 2D**). The cell margin of HUVECs co-cultured with DLBCL cells was rougher accompanied by more tiny protrusions. As proved in previous studies, EndMT was always companied with vascular inflammation; we therefore confirmed that both of the cell lines induced the production of inflammatory mediators (**Figures S2A, B**). In addition, SU-DHL-4 and OCI-LY3 cells increased the permeability of the endothelial cell layer, which indicated impaired structure and dysfunction of vascular endothelium (**Figure S2C**).

### Diffuse Large B-Cell Lymphoma Cells Prompted Proliferation, Extracellular Matrix Degradation, Migration, and Tube Formation of Human Umbilical Vein Endothelial Cells

**Figure 3:**
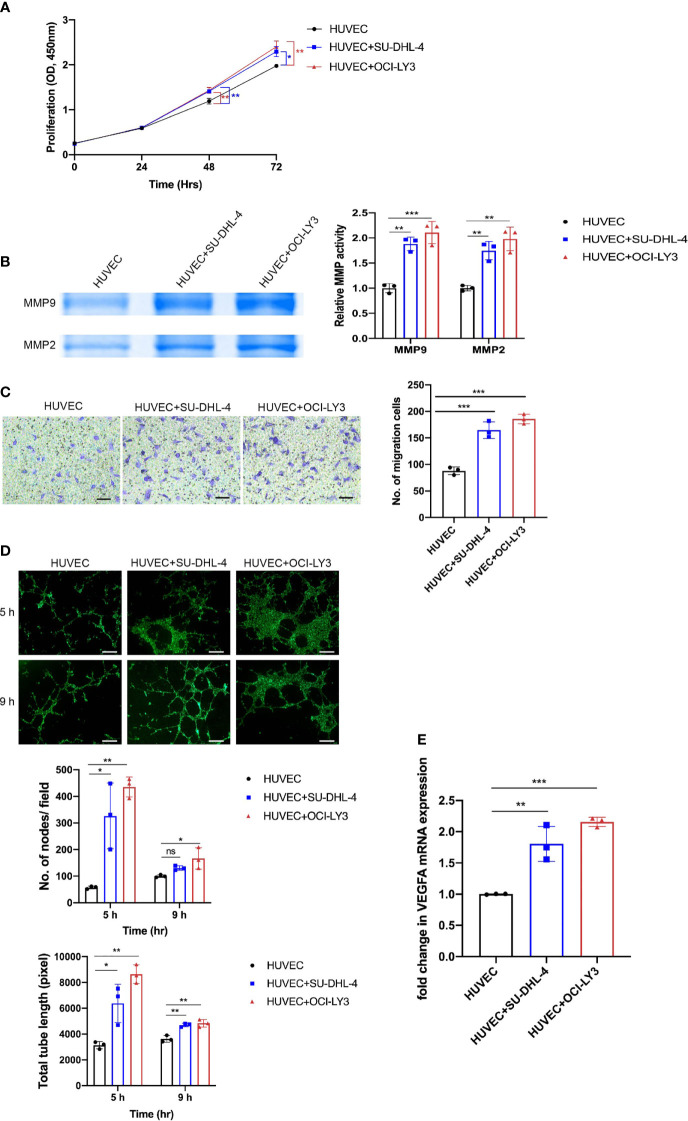
GCB and ABC DLBCL cells promoted proliferation, MMP2/9 activity, migration, and tube formation of endothelial cells. **(A)** Proliferation of endothelial cells was assessed by CCK-8 assay at indicated time points (24, 48, and 72 h). **(B)** Activity levels of MMP2/9 were evaluated by gelatin zymography. Representative images and quantitative analysis of MMP2/9 activity are shown. **(C)** Cell migration of HUVECs was detected by a transwell migration assay. Representative images and quantitative analysis of migratory cells are shown. Scale bar, 200 μm. **(D)** HUVECs seeded on Matrigel were co-cultured with or without DLBCL cells. Representative images of tube formation assay at 5 and 9 h were shown. Number of nodes per field and total tube length were quantified using ImageJ. Scale bar, 1 mm. **(E)** VEGF mRNA expression in HUVECs was analyzed by qRT-PCR. Error bars, SD; n = 3 independent experiments; *p < 0.05, **p < 0.01, ***p < 0.001. ns, not significant; GCB, germinal center B cell; ABC, activated B cell; DLBCL, diffuse large B-cell lymphoma; CCK-8, Cell Counting Kit-8; HUVEC, human umbilical vein endothelial cell.

Angiogenesis was a complex process that consists of multiple steps including proliferation, ECM degradation, migration, and tube formation. We evaluated the viability of HUVECs and found that SU-DHL-4 and OCI-LY3 cells were able to promote HUVEC proliferation after 48 and 72 h co-culture (**Figure 3A**). Additionally, HUVECs were observed with enhanced MMP2/9 enzymatic activities and migration potential after co-culture with SU-DHL-4 and OCI-LY3 cells (**Figures 3B, C**). Tube formation assay, which replicates multiple steps in angiogenesis, has been widely used as a screen for angiogenesis (27). In the tube formation experiment, HUVECs formed capillary-like tubular structures on Matrigel; quantitative analysis showed that the number of the nodes and total tube length significantly increased in the presence of SU-DHL-4 or OCI-LY3 cells (**Figure 3D**). Since VEGFA was a key pro-angiogenetic growth factor, SU-DHL-4 and OCI-LY3 cells also upregulated the mRNA level of VEGFA in HUVECs (**Figure 3E**). These results comprehensively demonstrated the angiogenetic phenotype changes GCB and ABC DLBCL cells made upon HUVECs.

### Human Umbilical Vein Endothelial Cells Undergoing Endothelial-to-Mesenchymal Transition Activated β-Catenin Signaling in Diffuse Large B-Cell Lymphoma Cells

After proving the capacity of DLBCL cells to induce EndMT in HUVECs, we further checked whether the acquisition of an EndMT profile by endothelial cells could in turn have any effect on DLBCL cells. It is well known that β-catenin signaling is associated with tumor cell growth, migration, invasion, and survival. Thus, we detected the activation of β-catenin signaling in DLBCL cells co-cultured with HUVECs undergoing EndMT in the present study. Immunoblot assays showed that co-culture with HUVECs undergoing EndMT robustly activated β-catenin signaling in both SU-DHL-4 and OCI-LY3, manifesting as increased protein levels of β-catenin and its downstream genes including c-myc and cyclin D1 (**Figure S3**). Considering the complex and distinctive effects that endothelial cells could make upon different kinds of DLBCL cells, more extensive functional experiments and mechanistic investigations are needed in further studies.

### Canonical WNT Signaling and Snail Were Involved in Diffuse Large B-Cell Lymphoma-Induced Endothelial-to-Mesenchymal Transition

**Figure 4:**
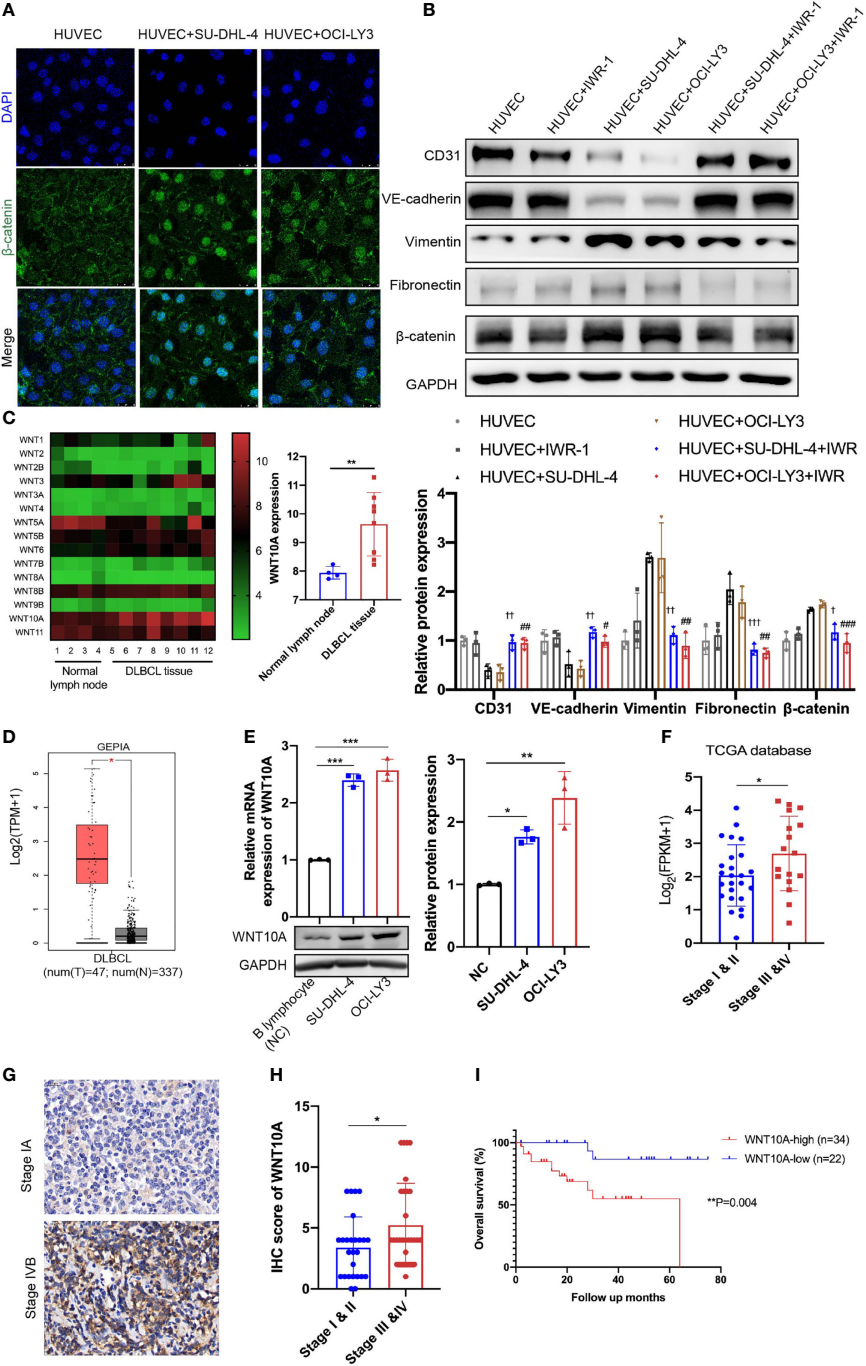
Canonical WNT signaling was involved in DLBCL-induced EndMT and WNT10A overexpression in DLBCL was associated with advanced stage and poor overall survival. **(A)** The subcellular localization of β-catenin in HUVECs was examined by immunofluorescence assay, and representative images are shown (n = 3 independent experiments). Scale bar, 25 μm. **(B)** 20 μM IWR-1 **(B)**, an inhibitor of the canonical WNT signaling, was added into HUVECs followed by incubating with DLBCL cells for 48 h, and the lysates of HUVECs were detected for endothelial and mesenchymal markers using immunoblot (n = 3 independent experiments). **(C)** Expression levels of WNT family members in normal lymph nodes (n = 4) and DLBCL tissues (n = 8) were detected by microarray analysis (left panel). Statistical analysis of WNT10A expression between normal lymph nodes (n = 4) and DLBCL tissues (n = 8) (right panel). **(D)** Differential mRNA expression profiles of WNT10A in DLBCL tissues and normal tissues were shown in Gene Expression Profiling Interactive Analysis database. **(E)** mRNA and protein expression of WNT10A in B lymphocytes from healthy donors and DLBCL cell lines were confirmed by qRT-PCR and immunoblot (n = 3 independent experiments). **(F)** High mRNA expression of WNT10A was correlated with advanced clinical stage in DLBCL according to The Cancer Genome Atlas (TCGA) database. Stage I and II (n = 25) and Stage III and IV (n = 17). **(G)** Representative immunohistochemistry staining of WNT10A in DLBCL tissues of early stage and advanced stage. Scale bar, 20 μm; n = 3 independent experiments. **(H)** Statistical analysis of WNT10A expression in DLBCL patients with different clinical stages. Stage I and II (n = 26) and Stage III and IV (n = 30). **(I)** Overall survival of DLBCL patients was analyzed by Kaplan–Meier analysis based on WNT10A expression. Error bars, SD; ^†^ *p* < 0.05, ^††^ *p* < 0.01, ^†††^ *p* < 0.001 vs. “HUVEC+SU-DHL-4”; ^#^ *p* < 0.05, ^##^ *p* < 0.01, ^###^ *p* < 0.001 vs. “HUVEC+OCI-LY3”; *p < 0.05, **p < 0.01, ***p < 0.001 vs. control. DLBCL, diffuse large B-cell lymphoma; EndMT, endothelial-to-mesenchymal transition; HUVEC, human umbilical vein endothelial cell.

After incubation with either DLBCL cells, HUVECs showed obvious upregulation of β-catenin (**Figure 2A**). Furthermore, immunofluorescence analysis showed enhanced nuclear accumulation of β-catenin (**Figure 4A**). In order to verify whether canonical WNT signaling is involved in DLBCL-induced EndMT, IWR-1 (an inhibitor of the canonical WNT signaling) was added into the co-culture system, and downregulation of β-catenin protein level proved that IWR-1 treatment worked in inhibiting the canonical WNT signaling. Results showed that IWR-1 alleviated SU-DHL-4/OCI-LY3-induced EndMT (**Figure 4B**). Knocking down snail using siRNA showed similar results, suggesting that SU-DHL-4/OCI-LY3-induced EndMT was snail-dependent (**Figure S4**). Taken together, these results indicated that canonical WNT signaling and snail performed vital roles in DLBCL-induced EndMT.

### WNT10A Was Overexpressed in Diffuse Large B-Cell Lymphoma and Predicted Poor Prognosis

Next, we examined differentially expressed genes between normal lymph nodes and DLBCL tissues using microarray analysis to identify which kind of WNT ligand mediates DLBCL-induced EndMT. Among WNT family members, the increase of WNT10A mRNA expression was most significant in DLBCL tissues compared with normal tissues (**Figure 4C**). The level of WNT10A mRNA expression was also analyzed using Gene Expression Profiling Interactive Analysis (GEPIA) database, showing a much higher WNT10A level in 47 DLBCL tissues than in 337 normal tissues (**Figure 4D**). qRT-PCR analyses and immunoblot further confirmed the expression of WNT10A in SU-DHL-4 and OCI-LY3. Relative mRNA expression and protein level of endogenous WNT10A in DLBCL cell lines SU-DHL-4 and OCI-LY3 were significantly higher than in B lymphocytes from healthy donors (negative control) (**Figure 4E**). Analyzing WNT10A expression in The Cancer Genome Atlas (TCGA) database indicated that elevated WNT10A expression in DLBCL patients was concerned with advanced clinical stages (III and IV) (**Figure 4F**). Consistent with data in the database, immunohistochemical staining also revealed WNT10A overexpression in DLBCL patients, and higher WNT10A expression was associated with advanced clinical stages and poor overall survival (**Figures 4G** **–I**).

### WNT10A Promoted Endothelial-to-Mesenchymal Transition in Human Umbilical Vein Endothelial Cells *via* GSK3β/β-Catenin/Snail Signaling

**Figure 5:**
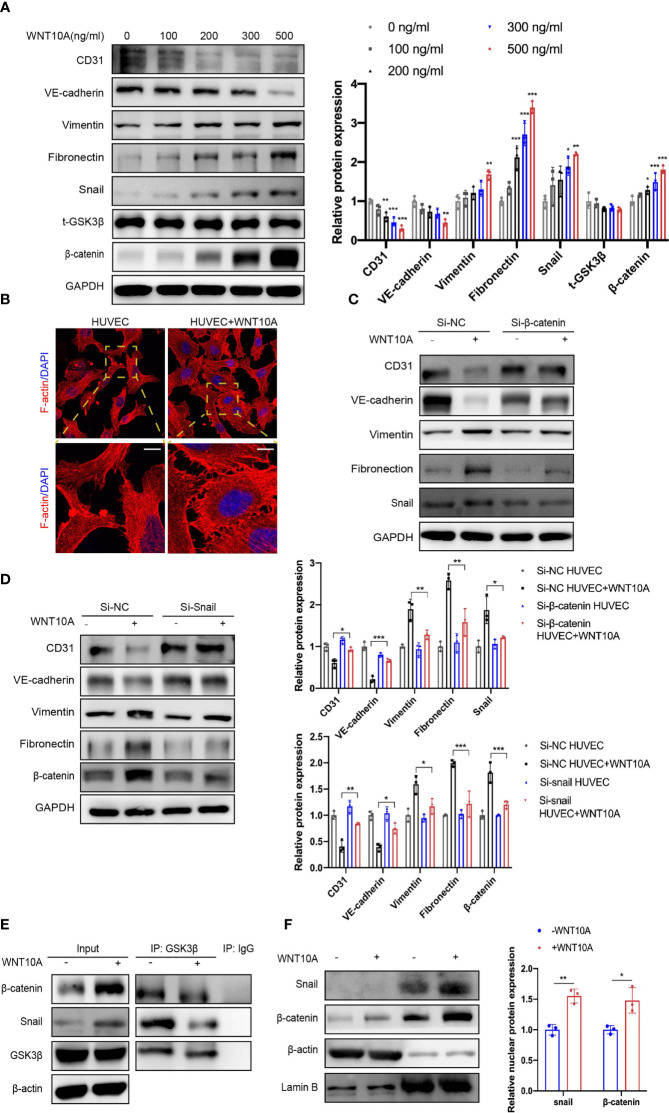
WNT10A prompted EndMT *via* GSK3β/β-catenin/snail signaling. **(A)** HUVECs were treated with different concentrations of WNT10A, and expression of EndMT markers was determined by immunoblot (n = 3 independent experiments). Error bars, SD. *p < 0.05, **p < 0.01, ***p < 0.001 vs. control. **(B)** The effects of WNT10A on the cytoskeleton rearrangement of HUVECs through F-actin staining. Scale bar, 10 μm; n = 3 independent experiments. **(C, D)** Immunoblot analysis of EndMT markers in control group and β-catenin **(C)** or snail **(D)** knockdown HUVECs with or without WNT10A treatment (n = 3 independent experiments). Error bars, SD. *p < 0.05, **p < 0.01, ***p < 0.001. **(E)** Co-IP analysis showed the interaction between GSK3β and β-catenin/snail in HUVECs. GSK3β, β-catenin, and snail were immunoprecipitated by antibodies against GSK3β. Antibody against IgG was used as a negative control. n = 3 independent experiments. **(F)** Cytoplasmic and nuclear extracts of HUVECs were separated. Protein levels of β-catenin and snail were analyzed by immunoblot. Cytoplasmic marker β-actin and nuclear membrane marker lamin B were used as loading controls (n = 3 independent experiments). Error bars, SD; *p < 0.05, **p < 0.01. EndMT, endothelial-to-mesenchymal transition; HUVEC, human umbilical vein endothelial cell; Co-IP, co-immunoprecipitation.

Based on the above clinical and experimental findings, we assumed DLBCL-derived WNT10A induces EndMT. To verify this assumption, we first treated HUVECs with different concentrations of rhWNT10A (**Figure 5A**). Following this treatment, 500 ng/ml of WNT10A markedly downregulated expressions of CD31 and VE-cadherin and upregulated the expressions of vimentin and FN, while some indicators were not significantly changed with lower concentrations of WNT10A. Thus, we chose 500 ng/ml as the concentration of WNT10A for the following experiments. In **Figure 5B**, cytoskeleton reorganization was observed in HUVECs after being treated with rhWNT10A. We further established WNT10A knockdown DLBCL cells to co-culture with HUVECs. As expected, depletion of WNT10A partially reduced DLBCL-induced EndMT, which could be rescued by exogenous WNT10A (**Figure S5**).

Considering the upregulation of β-catenin and snail in HUVECs treated with WNT10A, we further explored whether WNT10A-induced EndMT is β-catenin and snail-dependent. Knocking down β-catenin and snail in HUVECs treated with WNT10A restored the expression of CD31 and VE-cadherin and abrogated the upregulation of vimentin and FN (**Figures 5C, D**). Since no obvious protein expression change of GSK3β was observed in HUVECs treated with WNT10A (**Figure 5A**), we hypothesized that WNT10A acts through influencing the interaction between GSK3β and β-catenin or snail. We next performed Co-IP to verify the interaction between GSK3β and β-catenin/snail discovering WNT10A disrupted GSK3β/β-catenin/snail interactions in HUVECs, which promoted the stability of β-catenin and snail (**Figure 5E**). Immunoblot of nuclear protein suggested WNT10A prompted nuclear translocation of β-catenin and snail (**Figure 5F**).

### WNT10A Affected Human Umbilical Vein Endothelial Cell Proliferation, Extracellular Matrix Degradation, Migration, and Tube Formation *via* β-Catenin and Snail

**Figure 6:**
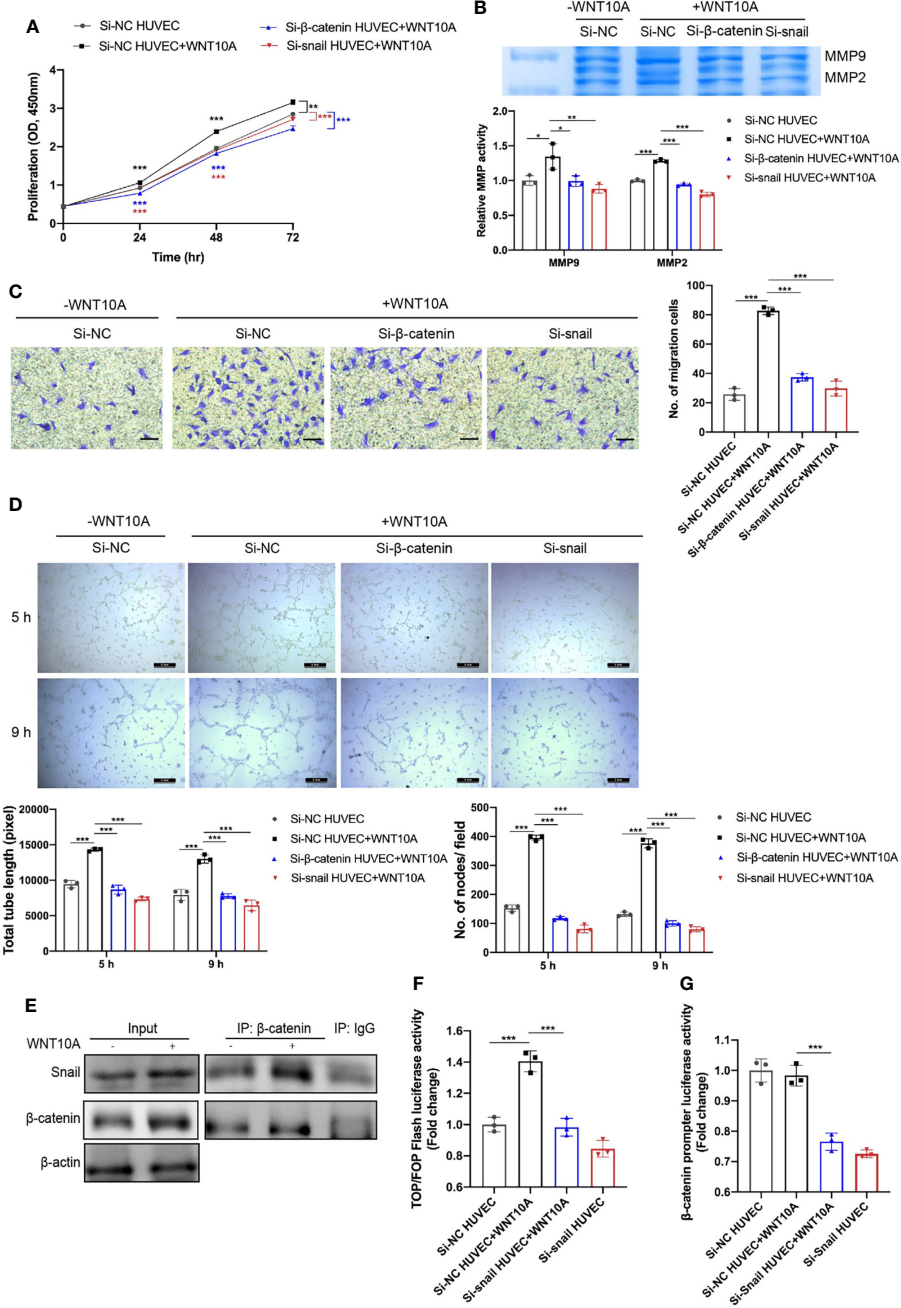
β-catenin and snail participated in WNT10A-induced proliferation, ECM degradation, migration, and tube formation ability, and β-catenin interacted with snail. **(A**–**D)** Cell proliferation **(A)**, MMP2/9 activity **(B)**, migration ability **(C)**, and tube formation ability **(D)** of HUVECs were evaluated by CCK-8 assay, gelatin zymography, transwell migration assay, and tube formation assay, respectively. HUVECs were transfected with negative control siRNA, β-catenin siRNA, or snail siRNA before WNT10A treatment. Scale bar, 200 μm. **(E)** Co-IP analysis showed the interaction between β-catenin and snail in HUVECs. β-catenin and snail were immunoprecipitated by antibodies against β-catenin. Antibody against IgG was used as a negative control. **(F)** β-Catenin transcriptional activity in control HUVECs and snail-silenced HUVECs were analyzed by Top/FopFlash reporter assay. **(G)** Activity of β-catenin promoter in control HUVECs and snail-silenced HUVECs were analyzed by dual-luciferase reporter assay. Error bars, SD; n = 3 independent experiments; *p < 0.05, **p < 0.01, ***p < 0.001. ECM, extracellular matrix; HUVEC, human umbilical vein endothelial cell; CCK-8, Cell Counting Kit-8; Co-IP, co-immunoprecipitation.

We next assessed the effects of WNT10A on angiogenesis and the underlying mechanisms. Enhanced proliferation, MMP2/9 enzymatic activities, and migration capability of HUVECS were detected after WNT10A treatment and can be blocked by β-catenin siRNA or snail siRNA (**Figures 6A** **–C**). Moreover, WNT10A treatment remarkably increased tube formation in HUVECs, which was also mediated by β-catenin and snail (**Figure 6D**), supporting the notion that WNT10A functions as an important mediator causing abnormal angiogenesis in DLBCL.

### β-Catenin Interacted With Snail

Both activations of β-catenin and snail in WNT10A-induced EndMT inspired us to investigate whether β-catenin and snail interact with each other. **Figure 5C** shows blocking β-catenin prevented WNT10A-induced snail upregulation, suggesting snail is a downstream gene of β-catenin. Therefore, Co-IP analysis was performed to identify whether β-catenin interacts with snail directly. We found WNT10A facilitated Co-IP of snail with β-catenin in lysates of HUVECs (**Figure 6E**). TopFlash reporter activity was enhanced by WNT10A, indicating that WNT10A enhanced the transcriptional activity of β-catenin (**Figure 6F**). Snail knockdown prevented WNT10A induced β-catenin-dependent transcription. Furthermore, a dual‐luciferase reporter assay was used to determine the activity of the β-catenin promoter. Silencing snail decreased β-catenin promoter activity, demonstrating that snail transcriptionally activates β-catenin (**Figure 6G**).

## Discussion

EndMT, one type of intricate cellular differentiation, shapes tumor microenvironment and favors tumorigenesis (7). It has been identified in the microenvironment of multiple solid malignancies including glioblastoma, pancreatic ductal adenocarcinoma, oral squamous cell carcinoma, and esophageal adenocarcinoma (28–31). Our study first proved robust endothelial plasticity in hematological malignancy. Immunofluorescence analysis demonstrated that endothelial cells in the DLBCL microenvironment have undergone EndMT, which was barely seen in healthy lymph nodes. Furthermore, EndMT markers may have potential in disease staging and prognosis prediction. In our serum exosome proteomics data, EndMT markers significantly differed between DLBCL patients with distinct clinical features and healthy donors. In addition, we confirmed the clinical significance of EndMT markers in a GEO database and revealed for the first time that mesenchymal markers vimentin and COLI were associated with overall survival in DLBCL patients. This finding supplies the previous conclusion that vimentin was overexpressed and contributed to cancer progression (32, 33). Although a larger sample size was needed to validate these findings, they provide insight into the development of potential new biomarkers in DLBCL for early diagnosis and prognosis prediction. In a study of pancreatic ductal adenocarcinoma, investigators proposed a potential EndMT index and revealed that a positive EndMT index was related to patients’ T4 staging (29). A similar EndMT index in DLBCL is needed for quantification of the EndMT level and confirmation of the association between EndMT and clinical characteristics.

Apart from *in vivo* study, we established an indirect EC and DLBCL cell co-culture model to verify if DLBCL cells contribute to the mesenchymal transition of ECs *in vitro*. SU-DHL-4 and OCI-LY3 cell lines selected in this study represented two main subtypes of DLBCL. Consistent with our hypothesis, DLBCL cells repressed the expression of endothelial markers CD31 and VE-cadherin and stimulated the expression of mesenchymal markers vimentin, FN, and α-SMA. Besides changes in EndMT markers, cells that have undergone EndMT were always accompanied by cytoskeleton reorganization, increased motility, and enhanced ECM degradation (7). In the present study, ECs were characterized by increased protrusions of lamellipodia and filopodia as well as actin filament formation after co-culture with DLBCL cells. Previous studies provided evidence that MMP2 and MMP9 triggered cell migration and invasion, and they were two major MMPs involved in tumor angiogenesis, increasing the bioavailability of VEGF (34). In our study, DLBCL cells enhanced the properties of migration and activity of MMP2/9 in HUVECs.

Accumulating evidence demonstrated the role of angiogenesis in lymphoma progression (35). During angiogenesis, a part of ECs, so-called “tip cells,” gained migration and invasion capability response to the angiogenic signal. These “tip cells” were followed by the so-called “stalk cells,” which proliferated and maintained the structure of nascent vessels (36). ECs involved in this process shared some EndMT features; thus, angiogenesis was deemed as a “partial EndMT.” A previous study unveiled colon cancer cells induced angiogenesis through induction of “partial EndMT” (37). Lymphangiogenesis in squamous cell carcinoma was accompanied by EndMT (30). Here we distinctly demonstrated that DLBCL cells prompted EndMT along with angiogenesis. Detection of EndMT offered thread for the mechanisms underlying increased angiogenesis and may provide new targets for antiangiogenic therapies in DLBCL.

It is worth noting that although GCB and ABC DLBCL were characterized by different mutation patterns, GCB and ABC DLBCL cell lines selected for the present study exerted similar effects on EC transdifferentiation described above including acquiring mesenchymal phenotypes and enhanced angiogenesis. Nevertheless, since there was only one type of GCB and ABC DLBCL cell lines applied in this study, further studies were needed for the suasive comparison between these two subtypes of DLBCL.

After verifying the influences DLBCL made upon endothelial cells, we further aimed to investigate the precise molecular mechanism. EndMT was triggered by various signals depending on specific cell types (8, 38, 39). Many investigations confirmed the role of the WNT pathway in EndMT and angiogenesis (39–42). WNT3B contributed to the EndMT in keloid pathogenesis. WNT5A/GSK3β/β-catenin signaling played a pivotal role in the angiogenesis of glioma-derived endothelial cells (41). Wang revealed that WNT5B modulated EndMT and lymphangiogenesis (30). In addition, snail, a key transcription factor, was dominantly reported for its pivotal role in EndMT and angiogenesis (43, 44). Consistent with these reports, our study demonstrated that canonical WNT signaling and snail were involved in DLBCL-induced EndMT.

Despite the extensive studies of various WNT gene expression profiles in different tumors, it was rarely investigated in DLBCL. Here, we screened the expression profile of the WNT family in DLBCL *via* microarray data and GEPIA database analyses, which demonstrated that WNT10A was significantly upregulated in DLBCL. However, less is known regarding the roles of WNT10A in hematological malignancies. Furthermore, we found a high expression of WNT10A predicted advanced clinical stage and poor prognosis. These data implied that WNT10A may play an important role in DLBCL progression.

To explore the novel mechanism that WNT10A signaling played in EndMT, we performed a series of experiments. Blocking WNT10A in DLBCL cell lines partially reversed WNT10A-induced EndMT and could be rescued by rhWNT10A. ECs treated with rhWNT10A acquired mesenchymal characteristics and enhanced angiogenesis capability, which could be attenuated by silencing β-catenin or snail. These results suggested DLBCL-derived WNT10A prompted angiogenesis and EndMT in a β-catenin/snail-dependent way. In addition, it is worth noting that DLBCL cells upregulated the expression of mesenchymal cell markers COLI and α-SMA, while WNT10A had no obvious influence on them. Thus, we suspected that other DLBCL-derived inducers may contribute to DLBCL-induced EndMT.

In the absence of WNT ligand, β-catenin is phosphorylated by a destruction complex consisting of GSK3β, axin, adenomatous polyposis coli (APC), and casein kinase 1α, followed by ubiquitin-proteasomal degradation (45). GSK3β also induces nuclear export and ubiquitylation of snail by phosphorylating it (15). The binding of WNT ligands to their receptor complexes results in the inactivation of GSK3β, increased stability, and nuclear translocation of snail as well as β-catenin (46–48). Consistent with previous studies, our study revealed that administration of WNT10A resulted in reduced binding of GSK3β to β-catenin/snail, causing nuclear accumulation of snail and β-catenin.

**Figure 7:**
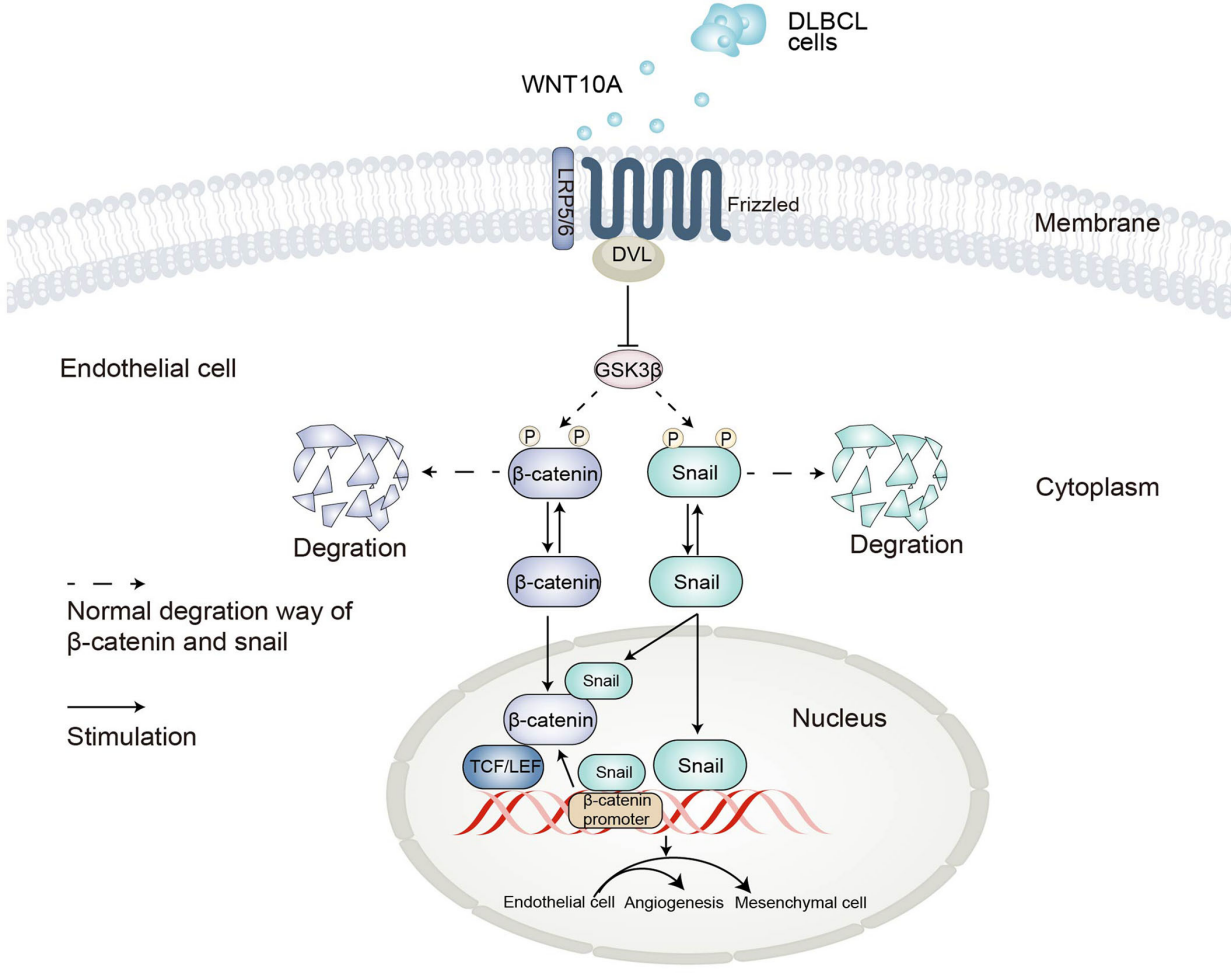
Schematic representation of the proposed mechanisms underlying DLBCL-induced angiogenesis and endothelial-to-mesenchymal transition. DLBCL, diffuse large B-cell lymphoma.

EndMT was a special form of Epithelial-mesenchymal transition (EMT) since the endothelial cell was a kind of epithelial cell (38). They shared similar processes and signaling pathways. A previous study reported β-catenin and snail-dependent signaling interacted during EMT, strengthening the robustness of two signaling ways (49). Our data proved that snail induced β-catenin-dependent transcriptional activity as well as the activity of the β-catenin promoter. On the other hand, WNT10A-induced snail upregulation was influenced by the protein level of β-catenin. For the first time, our research not only demonstrated the direct roles of β-catenin and snail in WNT10A-induced EndMT but also revealed the interconnection between these two crucial pathways activated by WNT10A. A schematic diagram of our hypothesis on the mechanisms of EndMT induced by DLBCL-derived WNT10A is shown in **Figure 7**.

To our knowledge, this is the first study that comprehensively sheds light on the existence, clinical relevance, phenotypic characteristics, and molecular mechanisms of DLBCL-associated EndMT and angiogenesis, revealing novel crosstalk between ECs and DLBCL cells in the lymphoma microenvironment. Nevertheless, in this study, we mainly focused on the effects of DLBCL on endothelial cells. Considering the significance of EndMT, which has been previously described in the progression of many other types of tumor, figuring out the involvement and impacts of EndMT on DLBCL can also make sense. Our further study will pay attention to the functional roles of EndMT during tumorigenesis, invasion, and metastasis. Especially, we will verify whether the *in vivo* inhibition of EndMT *via* suppressing WNT signaling impacts the progression of DLBCL. This will greatly enrich the results in the present study and may provide WNT10A/GSK3β/β-catenin/snail axis as a potentially effective target for anti-DLBCL therapy.

## Data Availability Statement

Publicly available datasets were analyzed in this study. These data can be found here: http://gepia.cancer-pku.cn/index.html, https://portal.gdc.cancer.gov, and https://www.ncbi.nlm.nih.gov/gds/?term=.

## Ethics Statement

The studies involving human participants were reviewed and approved by Renji Hospital Ethics Committee, School of Medicine, Shanghai Jiaotong University. The patients/participants provided their written informed consent to participate in this study.

## Author Contributions

XS designed the experiments, performed the majority of the experiments, analyzed the data, and wrote the draft. TW is the corresponding author of the manuscript, helped conceive the study, designed the experiments, supervised the study, and modified the manuscript. JF assisted in the immunohistochemical assessment. SZ and FY both contributed to the sample collection, exosome isolation and quantitative proteomics of exosomes. FY also participated in cell culture. All the authors reviewed the article and approved the final version.

## Funding

This research was funded by the National Natural Science Foundation of China (No. 81570177).

## Conflict of Interest

The authors declare that the research was conducted in the absence of any commercial or financial relationships that could be construed as a potential conflict of interest.

## Publisher’s Note

All claims expressed in this article are solely those of the authors and do not necessarily represent those of their affiliated organizations, or those of the publisher, the editors and the reviewers. Any product that may be evaluated in this article, or claim that may be made by its manufacturer, is not guaranteed or endorsed by the publisher.
