# Supplementary figures and images for: Diffuse Large B-Cell Lymphoma Promotes Endothelial-to-Mesenchymal Transition via WNT10A/Beta-Catenin/Snail Signaling
Source: Front Oncol. 2022 Apr 12;12:871788. doi: 10.3389/fonc.2022.871788 (PMC9039659; doi:10.3389/fonc.2022.871788)

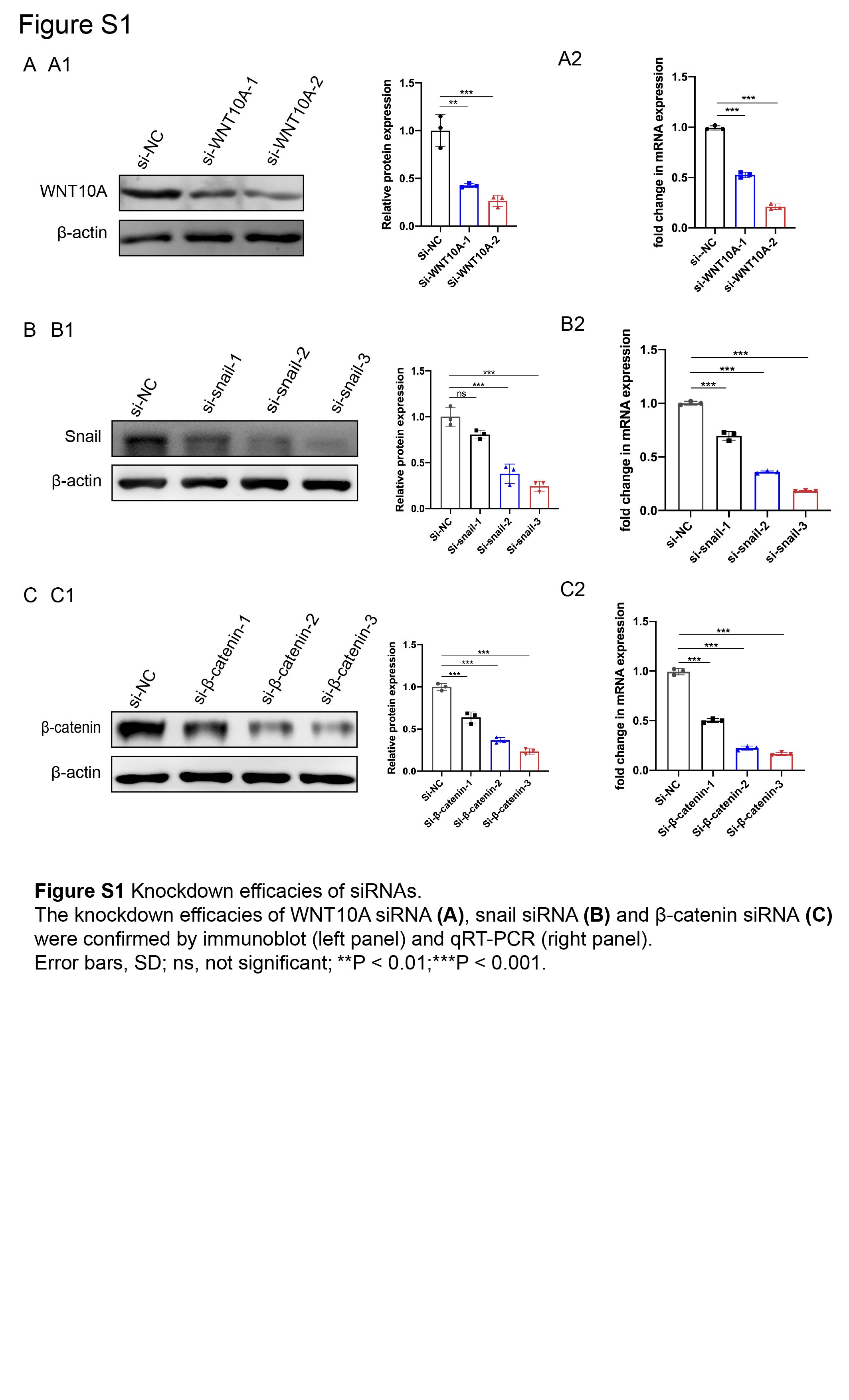

Supplement: Supplementary file 1 [file Image_1.jpg]

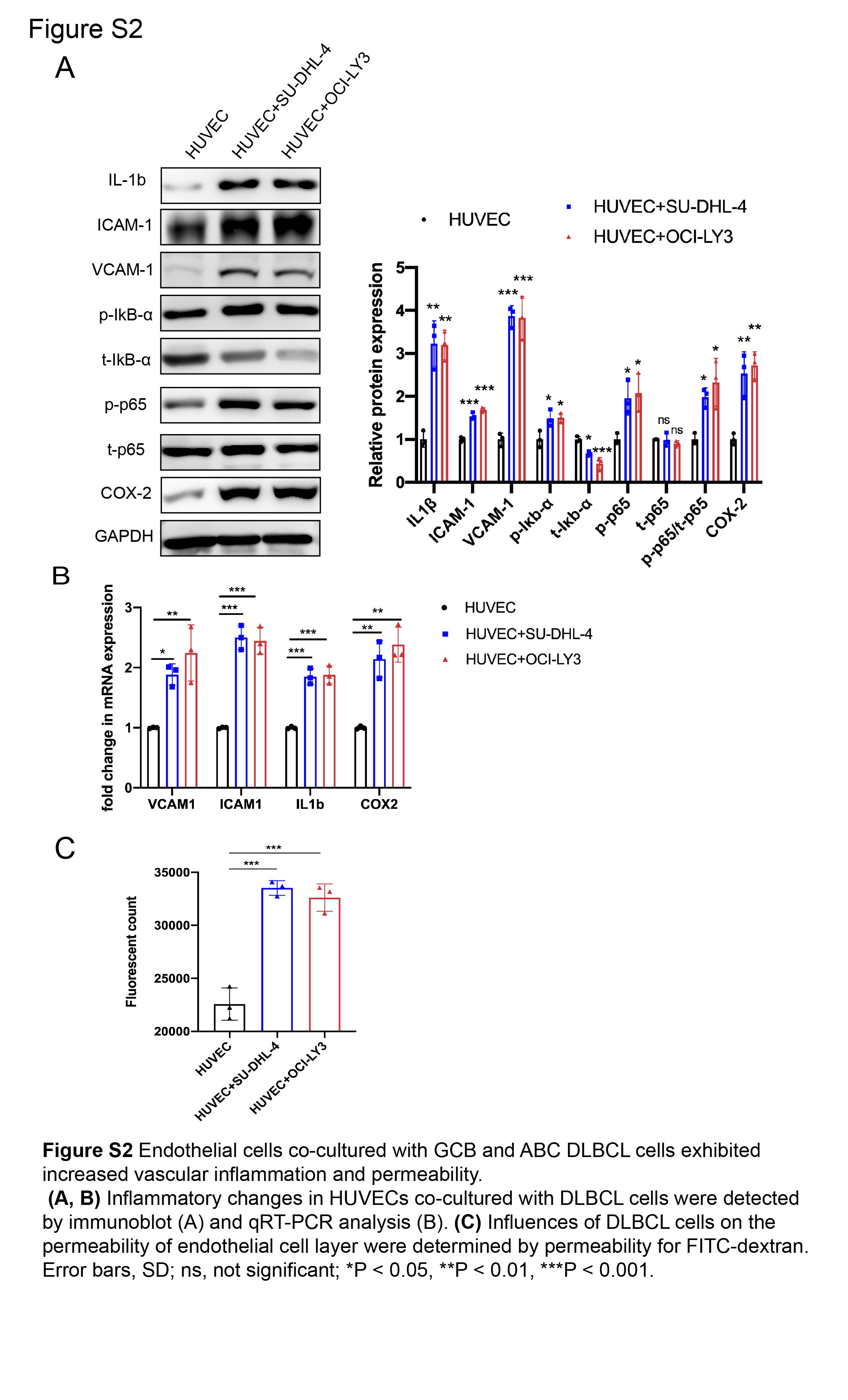

Supplement: Supplementary file 2 [file Image_2.jpg]

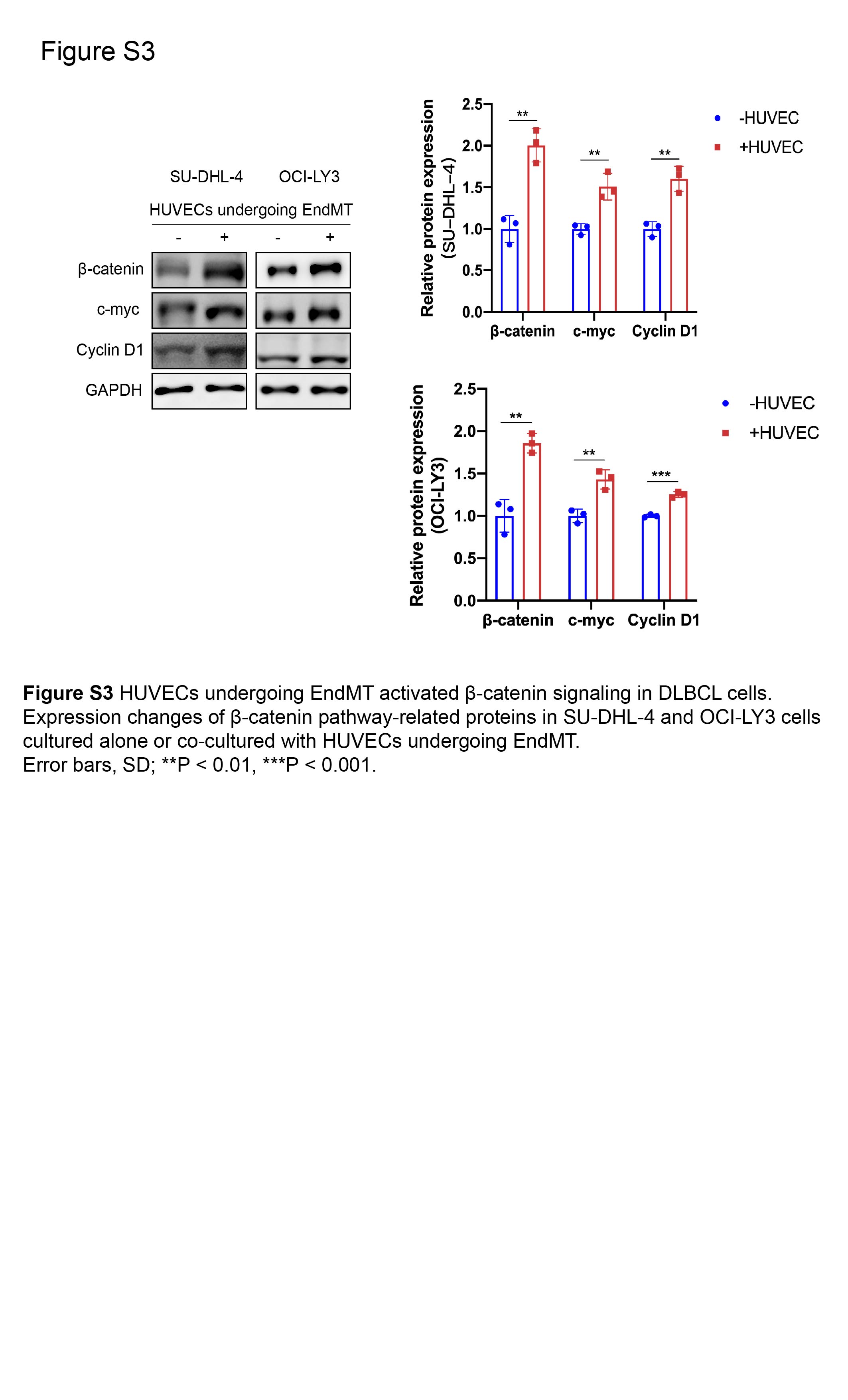

Supplement: Supplementary file 3 [file Image_3.jpg]

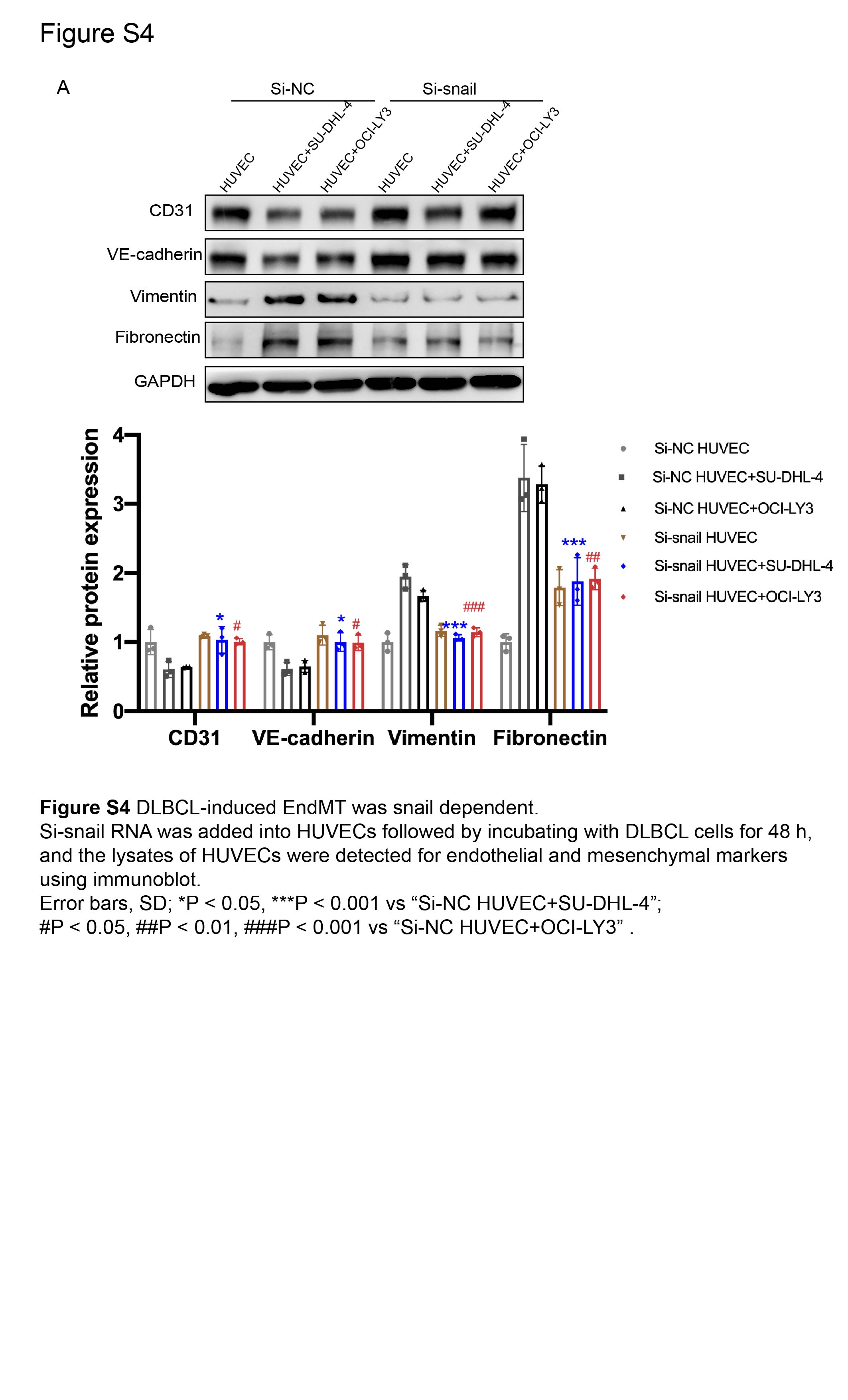

Supplement: Supplementary file 4 [file Image_4.jpg]

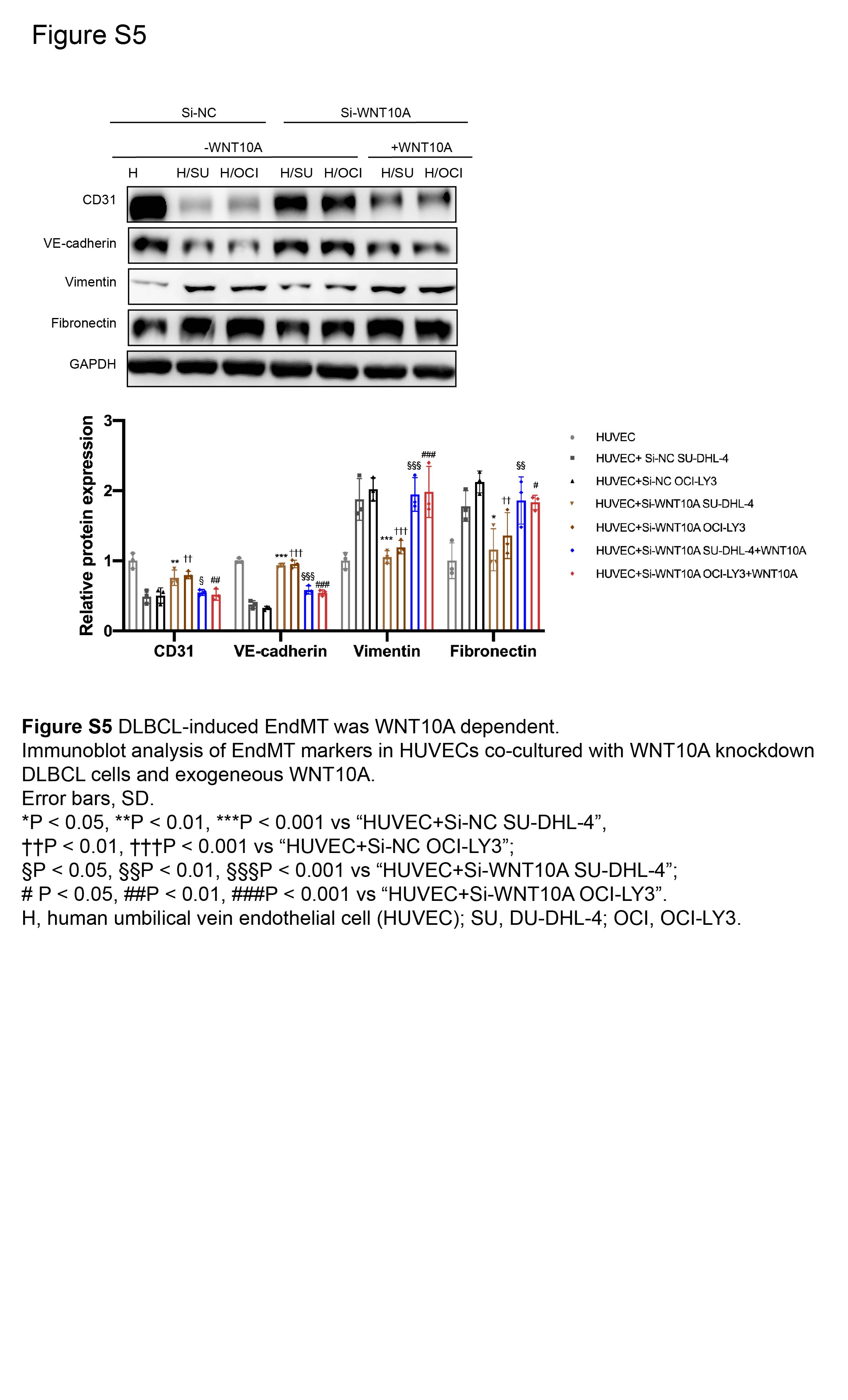

Supplement: Supplementary file 5 [file Image_5.jpg]
