# Supplementary material for: Diffuse Large B-Cell Lymphoma Promotes Endothelial-to-Mesenchymal Transition via WNT10A/Beta-Catenin/Snail Signaling
Source: Front Oncol. 2022 Apr 12;12:871788. doi: 10.3389/fonc.2022.871788 (PMC9039659; doi:10.3389/fonc.2022.871788)
Supplement: Supplementary file 6 [file Table_1.docx]

**Table S1.** List of primer sequences used for qRT-PCR assays in this study.

| **Name** | **Sequences** |
| --- | --- |
| PECAM-1-F | 5′-AACAGTGTTGACATGAAGAGCC -3′ |
| PECAM-1-R | 5′- TGTAAAACAGCACGTCATCCTT-3′ |
| CDH5-F | 5′-GTTCACGCATCGGTTGTTCAA-3′ |
| CDH5 -R | 5′-CGCTTCCACCACGATCTCATA-3′ |
| VIM-F | 5′-CGTGAATACCAAGACCTGCTC-3′ |
| VIM-R | 5′-GGAAAAGTTTGGAAGAGGCAG-3′ |
| FN1-F | 5′-CCATCGCAAACCGCTGCCAT -3′ |
| FN1-R | 5′- AACACTTCTCAGCTATGGGCTT-3′ |
| ACTA2-F | 5′-AATGCAGAAGGAGATCACGG-3′ |
| ACTA2-R | 5′-TCCTGTTTGCTGATCCACATC-3′ |
| COLⅠAⅠ-F | 5′- GAGGGCCAAGACGAAGACATC-3′ |
| COLⅠAⅠ-R | 5′- CAGATCACGTCATCGCACAAC-3′ |
| SNAI1-F | 5′- TCGGAAGCCTAACTACAGCGA-3′ |
| SNAI1-R | 5′- AGATGAGCATTGGCAGCGAG-3′ |
| ZEB1-F | 5′-GATGATGAATGCGAGTCAGATGC-3′ |
| ZEB1-R | 5′-ACAGCAGTGTCTTGTTGTTGT-3′ |
| ZEB2-F | 5′-GGAGACGAGTCCAGCTAGTGT-3′ |
| ZEB2-R | 5′-CCACTCCACCCTCCCTTATTT-3′ |
| TWIST1-F | 5′-CCGGAGACCTAGATGTCATTG-3′ |
| TWIST1-R | 5′-CCACGCCCTGTTTCTTTG-3′ |
| VCAM1-F | 5′-TTTGACAGGCTGGAGATAGACT-3′ |
| VCAM1-R | 5′-TCAATGTGTAATTTAGCTCGGCA-3′ |
| ICAM1-F | 5′-ATGCCCAGACATCTGTGTCC-3′ |
| ICAM1-R | 5′-GGGGTCTCTATGCCCAACAA-3′ |
| IL1b-F | 5′-CGATCACTGAACTGCACGCTC-3′ |
| IL1b-R | 5′-TTATATCCTGGCCGCCTTTGG-3′ |
| COX2-F | 5′-CCCTTGGGTGTCAAAGGTAA-3′ |
| COX2-R | 5′-GCCCTCGCTTATGATCTGTC-3′ |
| ACTB-F | 5′-TGGCACCCAGCACAATGAA-3′ |
| ACTB-R | 5′-CTAAGTCATAGTCCGCCTAGAAGCA-3′ |

Gene name versus protein name: PECAM-1 vs. Platelet endothelial cell adhesion molecule (CD31); CDH5 vs. VE-cadherin; VIM vs. Vimentin; FN1 vs. Fibronectin; ACTA2 vs. α-SMA; SNAI1 vs. Snail; ACTB vs. β-actin
