# Supplementary material for: Diffuse Large B-Cell Lymphoma Promotes Endothelial-to-Mesenchymal Transition via WNT10A/Beta-Catenin/Snail Signaling
Source: Front Oncol. 2022 Apr 12;12:871788. doi: 10.3389/fonc.2022.871788 (PMC9039659; doi:10.3389/fonc.2022.871788)
Supplement: Supplementary file 7 [file Table_2.docx]

Table S2: The target sequences of siRNAs used in our study.

| **Name** | **Sequences** |  |
| --- | --- | --- |
| si-WNT10A-1 | 5′-CCACGAATGCCAACACCAA-3′ |  |
| si-WNT10A-2^*^ | 5′-GGTCAGCACCCAATGACAT-3′ |  |
| si-snail-1 | 5′-CAGGCUCGAAAGGCCUUCA-3′ |  |
| si-snail-2 | 5′-GCUCUUUCCUCGUCAGGAA-3′ |  |
| si-snail-3^*^ | 5′-CGGACCUUCUCCCGAAUGU-3′ |  |
| si-β-catenin-1 | 5′-CCUUCACUAUGGACUACCA-3′ |  |
| si-β-catenin-2 | 5′-CGGGAUGUUCACAACCGAA-3′ |  |
| si-β-catenin-3^*^ | 5′-GGGUAGGGUAAAUCAGUAA-3′ |  |

Sequences with ‘^*^’ were chosen in this study.
